# The Independent Association of TSH and Free Triiodothyronine Levels With Lymphocyte Counts Among COVID-19 Patients

**DOI:** 10.3389/fendo.2021.774346

**Published:** 2022-01-13

**Authors:** David Tak Wai Lui, Chi Ho Lee, Wing Sun Chow, Alan Chun Hong Lee, Anthony Raymond Tam, Polly Pang, Tip Yin Ho, Chloe Yu Yan Cheung, Carol Ho Yi Fong, Chun Yiu Law, Kelvin Kai Wang To, Ching Wan Lam, Kathryn Choon Beng Tan, Yu Cho Woo, Ivan Fan Ngai Hung, Karen Siu Ling Lam

**Affiliations:** ^1^ Department of Medicine, The University of Hong Kong, Queen Mary Hospital Hong Kong, Hong Kong SAR, China; ^2^ Division of Chemical Pathology, Queen Mary Hospital Hong Kong, Hong Kong SAR, China; ^3^ Department of Microbiology, The University of Hong Kong, Queen Mary Hospital Hong Kong, Hong Kong SAR, China; ^4^ Department of Pathology, The University of Hong Kong Hong Kong, Hong Kong SAR, China

**Keywords:** COVID-19, SARS-CoV-2, thyroid function tests, lymphopenia, lymphocytes, euthyroid sick syndromes

## Abstract

**Background:**

Both lymphopenia and thyroid dysfunction are commonly observed among COVID-19 patients. Whether thyroid function independently correlates with lymphocyte counts (LYM) remains to be elucidated.

**Methods:**

We included consecutive adults without known thyroid disorder admitted to Queen Mary Hospital for COVID-19 from July 2020 to April 2021 who had thyroid-stimulating hormone (TSH), free thyroxine (fT4), free triiodothyronine (fT3) and LYM measured on admission.

**Results:**

A total of 541 patients were included. Median LYM was 1.22 x 10^9^/L, with 36.0% of the cohort lymphopenic. 83 patients (15.4%) had abnormal thyroid function tests (TFTs), mostly non-thyroidal illness syndrome (NTIS). Patients with lymphopenia had lower TSH, fT4 and fT3 levels than those without. Multivariable stepwise linear regression analysis revealed that both TSH (standardized beta 0.160, p<0.001) and fT3 (standardized beta 0.094, p=0.023), but not fT4, remained independently correlated with LYM, in addition to age, SARS-CoV-2 viral load, C-reactive protein levels, coagulation profile, sodium levels and more severe clinical presentations. Among the 40 patients who had reassessment of TFTs and LYM after discharge, at a median of 9 days from admission, there were significant increases in TSH (p=0.031), fT3 (p<0.001) and LYM (p<0.001). Furthermore, patients who had both lymphopenia and NTIS were more likely to deteriorate compared to those who only had either one alone, and those without lymphopenia or NTIS (p for trend <0.001).

**Conclusion:**

TSH and fT3 levels showed independent positive correlations with LYM among COVID-19 patients, supporting the interaction between the hypothalamic-pituitary-thyroid axis and immune system in COVID-19.

## Introduction

Lymphopenia is a common hematologic finding in coronavirus disease 2019 (COVID-19) (1), carrying prognostic implication in view of its association with disease severity and mortality (2). On the other hand, thyroid involvement by COVID-19 is increasingly recognized since the first report of subacute thyroiditis after COVID-19 (3). Data from larger cohorts of COVID-19 patients have enabled better delineation of the patterns of thyroid dysfunction, which include thyroiditis and non-thyroidal illness (NTIS) (4). NTIS, characterized by low free triiodothyronine (fT3) levels, also carries prognostic implication in COVID-19 (5–7). Furthermore, patients with more severe illness were reported to have concomitant low thyroid-stimulating hormone (TSH) levels (6, 7). These highlight the clinical relevance of lymphocyte counts, TSH and thyroid hormones in the course of COVID-19. Studies have suggested potential effects of TSH and thyroid hormones on the immune system, including the lymphocyte population (8). For example, animal studies have suggested the potential role of TSH in improving lymphocyte proliferation (9); circulating thyroid hormone levels are positively associated with immunological reactivity among healthy individuals, such as maintenance of the lymphocyte subpopulations (10).

In COVID-19, postulated mechanisms for lymphopenia include: (i) the direct effect of severe acute respiratory syndrome coronavirus 2 (SARS-CoV-2) on the apoptosis of lymphocytes, bone marrow impairment and thymic suppression; (ii) cytokine-induced apoptosis of lymphocytes; and (iii) metabolic and biochemical derangements (such as lactic acidosis) (11) which may influence the production, survival and function of lymphocytes (2). In fact, all these postulated mechanisms may also lead to the disturbances in the hypothalamic-pituitary-thyroid axis, previously reported in COVID-19 patients (7, 12, 13). COVID-19 may also involve multiple extrapulmonary systems, as SARS-CoV-2 entry receptor – angiotensin converting enzyme 2 (ACE2) – expression is found in a wide variety of human tissues (14). A recent study suggested potential associations between TSH/thyroid hormones and lymphopenia in a Dutch cohort of COVID-19 patients, showing that patients with severe lymphopenia had lower TSH, free thyroxine (fT4) and fT3 levels and higher levels of inflammatory markers, similar to findings in patients with bacterial sepsis (15). Whether this association is an epiphenomenon confounded by the presence of all the above discussed factors remains to be elucidated. Hence, we carried out this study to investigate whether an independent association exists between TSH/thyroid hormones and lymphopenia in COVID-19 patients, which may shed light onto the interaction of TSH/thyroid hormones with the immune system in the clinical course of COVID-19.

## Methods

Public health ordinance in Hong Kong required all patients tested positive for COVID-19 be admitted to hospital (16), including those detected on contact tracing and Universal Community Testing Programme (17), regardless of symptoms. Our institution, Queen Mary Hospital, is one of the major centers in Hong Kong receiving confirmed COVID-19 patients. Consecutive adult patients (aged ≥18 years) admitted to Queen Mary Hospital for COVID-19 between 21 July 2020 and 20 April 2021 were prospectively recruited (12, 18, 19). The presence of SARS-CoV-2 was confirmed in all patients by RT-PCR from the nasopharyngeal swab (NPS) and/or deep throat saliva (DTS), using the LightMix SarbecoV E-gene assay (TIB Molbiol, Berlin, Germany) which targeted the envelope protein (E) gene of SARS-CoV-2 as we described previously (20). Patients were excluded if they (i) had history of thyroid, pituitary or hypothalamic disorders; (ii) were on anti-thyroid drugs or thyroid hormone replacement; (iii) were on medications with potential impact on thyroid function including systemic steroid, amiodarone, heparin and dopamine; or (iv) had active hematologic or solid malignancies. Each patient had blood tests within 24 hours after admission, before the initiation of COVID-19 treatments.

Serum TSH, fT4 and fT3 were measured with immunoassays ADVIA Centaur^®^ TSH3-Ultra, FT4 and FT3 assays respectively (Siemens Healthcare Diagnostics Inc., USA). The reference ranges for TSH, fT4 and fT3 were 0.35–4.8 mIU/L, 12–23 pmol/L and 3.2–6.5 pmol/L, respectively. Anti-thyroglobulin (anti-Tg) and anti-thyroid peroxidase (anti-TPO) antibody titers were measured with QUANTA Lite^®^ Thyroid T and TPO enzyme-linked immunosorbent assay respectively (Inova Diagnostics, USA). Positive anti-Tg and anti-TPO was defined by >100 units. Anti-TSH receptor antibody (anti-TSHR) titer was measured with Anti-TSH Receptor (TRAb) Fast ELISA (IgG) test kit (EUROIMMUN Medizinische Labordiagnostika AG, Germany), using porcine TSHR. Anti-TSHR was considered positive if >1 IU/L. NTIS was defined by low fT3 with normal/low TSH (21).

Basic hematology and biochemistry panel, glycated hemoglobin (HbA1c) and C-reactive protein (CRP) were measured. Lymphopenia was defined according to the laboratory reference range, i.e. if absolute lymphocyte count <1.06×10^9^/L. Estimated glomerular filtration rate (eGFR) was calculated using the Chronic Kidney Disease Epidemiology Collaboration (CKD-EPI) equation in all individuals (22). Abnormalities in the hematological and biochemical parameters were defined by their respective laboratory reference ranges.

Demographics and major comorbidities were recorded. Obesity was defined by the International Classification of Diseases, Ninth Revision, Clinical Modification (ICD-9-CM) code 278.0. Diabetes was defined by a known diagnosis of diabetes or HbA1c ≥6.5% on admission. COVID-19-related symptoms were evaluated with a standard checklist. Respiratory rate, baseline oxygen saturation by pulse oximetry, and oxygen requirement on admission were captured. Chest x-ray was performed in each patient on admission. Cycle threshold (Ct) values were obtained from the qualitative LightMix SarbecoV E-gene assay (TIB Molbiol, Berlin, Germany) performed on specimens from NPS and/or DTS (whichever was lower) on admission. The Ct value represents the number of cycles required for a gene target or a PCR product to be detected. While viral loads were not directly measured with a dedicated quantitative RT-PCR assay in this analysis, studies have shown a good correlation between Ct values and SARS-CoV-2 viral loads (23, 24), such that the lower the Ct values, the higher the viral loads.

COVID-19 severity was classified into mild, moderate, severe and critical according to the ‘Chinese Clinical Guidance for COVID-19 Pneumonia Diagnosis and Treatment (7^th^ edition)’ published by the Chinese National Health Commission (NHC) (25). Each patient’s clinical outcomes were captured. Severe COVID-19 outcomes were defined by a composite of new-onset oxygen requirements, intubation and mechanical ventilation, intensive care unit (ICU) admission and death.

In the early phase of this study, reassessment blood tests including thyroid function tests (TFTs) and lymphocyte counts were arranged around 1–2 weeks after discharge. Due to the subsequent significant increase in the case load of COVID-19 patients, the early reassessment was discontinued. Hence, only a subset of patients had reassessment of TFTs and lymphocyte counts early after discharge.

The study followed the principles in the Declaration of Helsinki and was approved by the Institutional Review Board of the University of Hong Kong/Hospital Authority Hong Kong West Cluster. All participants gave informed consent.

All statistical analyses were performed with IBM^®^ SPSS^®^ version 26. Two-sided p-values <0.05 were considered statistically significant. Data were presented as median with interquartile range (IQR), or number with percentage as appropriate. Data not conforming to normal distributions were logarithmically transformed before analyses. Between-group comparisons were performed with t-test for continuous variables, and Chi-square or Fisher’s exact test for categorical variables as appropriate. Pearson correlation was used to analyze the univariate correlation between clinical variables and lymphocyte counts. Multivariable stepwise linear regression analysis was used to identify the independent variables associated with lymphocyte counts. Multivariable stepwise logistic regression analysis was used to identify the independent variables associated with severe COVID-19 outcomes. All variables with statistical significance in the univariate analysis were included in the multivariable regression analysis.

Several sensitivity analyses were performed in the evaluation of the associations between TFTs and lymphocyte counts: (i) repeating the analyses after excluding patients with overt/subclinical hypothyroidism and overt thyrotoxicosis; and (ii) evaluating the correlation between TSH and lymphocyte counts in the subgroup of patients with low fT3.

## Results

In total, 541 patients were included in this analysis. Median age was 50 years (IQR: 36 – 63) and 245 (45.3%) were men. Their baseline characteristics are summarized in **Table 1**. Hypertension (21.1%) and diabetes (16.1%) were the most common comorbidities. Most patients (n=380, 70.2%) were symptomatic at presentation: cough, fever and sore throat were the most common symptoms. Most patients had non-severe disease on presentation, only 3.1% of the cohort required supplementary oxygen on admission. Only 1.5% of the cohort carried the SARS-CoV-2 variants, including mutations N501Y, L452R and E484K. The median lymphocyte count on admission was 1.22 x 10^9^/L (IQR: 0.90 – 1.68), with 36.0% of the cohort being lymphopenic.

**Table 1:** Baseline characteristics of the cohort.

	All	Normal Lymphocyte Count	Lymphopenia	Age-Adjusted P value
Number	541	346	195	---
Age (years)	50.0 (36.0 – 63.0)	46.0 (34.0 – 61.0)^a^	57.0 (42.0 – 66.0)^a^	---
Male	245 (45.3%)	148 (42.8%)	97 (49.7%)	0.076
**Thyroid function test**				
TSH (mIU/L)	1.20 (0.78 – 1.70)	1.30 (0.91 – 1.80)	1.00 (0.61 – 1.50)	**<0.001**
fT4 (pmol/L)	17.0 (15.0 – 19.0)	18.0 (16.0 – 19.0)	17.0 (15.0 – 18.0)	**0.025**
fT3 (pmol/L)	4.2 (3.7 – 4.8)	4.4 (4.0 – 4.9)	3.9 (3.4 – 4.4)	**<0.001**
**Comorbidities**				
Hypertension	114 (21.1%)	64 (18.5%)	50 (25.6%)	0.463
Diabetes	87 (16.1%)	51 (14.7%)	36 (18.5%)	0.376
Obesity	26 (4.8%)	16 (4.6%)	10 (5.1%)	0.955
IHD/CHF	22 (4.1%)	14 (4.0%)	8 (4.1%)	0.160
Stroke/TIA	13 (2.4%)	4 (1.2%)	9 (4.6%)	0.153
Cancer	17 (3.1%)	7 (2.0%)	10 (5.1%)	0.281
**Symptomatic presentation**	380 (70.2%)	218 (63.0%)	162 (83.1%)	**<0.001**
Fever	180 (33.3%)	95 (27.5%)	85 (43.6%)	**<0.001**
Myalgia	58 (10.7%)	37 (10.7%)	21 (10.8%)	0.966
Malaise	69 (12.8%)	37 (10.7%)	32 (16.4%)	0.079
Rhinorrhoea	66 (12.2%)	43 (12.4%)	23 (11.8%)	0.903
Cough	218 (40.3%)	126 (36.4%)	92 (47.2%)	0.088
Dyspnoea	33 (6.1%)	17 (4.9%)	16 (8.2%)	0.256
Sore throat	135 (25.0%)	79 (22.8%)	56 (28.7%)	0.064
Headache	56 (10.4%)	32 (9.2%)	24 (12.3%)	0.102
Nausea/vomiting	19 (3.5%)	14 (4.0%)	5 (2.6%)	0.384
Diarrhoea	59 (10.9%)	43 (12.4%)	16 (8.2%)	0.088
Anosmia/ageusia	63 (11.6%)	44 (12.7%)	19 (9.7%)	0.617
**Symptom count ≥3**	163 (30.1%)	99 (28.6%)	64 (32.8%)	0.320
**Viral load**				
Ct value at baseline	24.76 (18.01 – 31.20)	27.50 (19.01 – 33.00)	21.00 (16.70 – 26.32)	**<0.001**
**Acute phase reactants**				
C-reactive protein (mg/dL)	0.57 (0.31 – 2.05)	0.39 (0.31 – 1.39)	1.06 (0.31 – 3.14)	**<0.001**
Albumin (g/L)	42.0 (40.0 – 45.0)	43 (41 – 46)	42 (39 – 44)	**0.040**
**Coagulation profile**				
Platelet (x 10^9^/L)	217 (174 – 266)	236 (190 – 284)	191 (156 – 225)	**<0.001**
Prothrombin time (s)	11.7 (11.4 – 12.1)	11.6 (11.3 – 12.0)	11.9 (11.6 – 12.3)	**<0.001**
**Biochemical parameters**				
Sodium (mmol/L)	140 (138 – 141)	140 (138 – 141)	139 (137 – 140)	**<0.001**
Potassium (mmol/L)	3.7 (3.4 – 4.0)	3.8 (3.5 – 4.0)	3.7 (3.4 – 4.0)	0.054
Urea (umol/L)	3.9 (3.1 – 4.8)	3.9 (3.0 – 4.7)	4.1 (3.2 – 5.0)	0.739
eGFR (mL/min)	96 (82 – 109)	98 (88 – 112)	91.2 (75.4 – 103.0)	0.237
ALT (U/L)	25 (17 – 39)	26 (18 – 40)	22 (17 – 35)	0.278
AST (U/L)	27 (21 – 37)	27 (21 – 35)	28 (22 – 40)	0.078
LDH (U/L)	212 (179 – 263)	212 (180 – 260)	211 (179 – 267)	0.525
Creatine kinase (U/L)	98 (67 – 155)	95 (66 – 149)	108 (69 – 160)	0.176
Troponin T (ng/L)	5.71 (3.78 – 8.42)	5.56 (3.61 – 7.72)	6.23 (4.31 – 9.65)	0.354
**Oxygen requirement on admission**	17 (3.1%)	5 (1.4%)	12 (6.2%)	**0.016**

Data are presented as median (interquartile range) and number (percentage) as appropriate.

Values in bold represent statistical significance.

a p < 0.001 in the comparison of age among patients with normal lymphocyte counts and lymphopenia.

TSH, thyroid stimulating hormone; fT4, free thyroxine; fT3, free triiodothyronine; IHD, ischaemic heart disease; CHF, congestive heart failure; TIA, transient ischaemic attack; eGFR, estimated glomerular filtration rate; ALT, alanine aminotransferase; AST, aspartate aminotransferase; LDH, lactate dehydrogenase.

**Figure 1:**
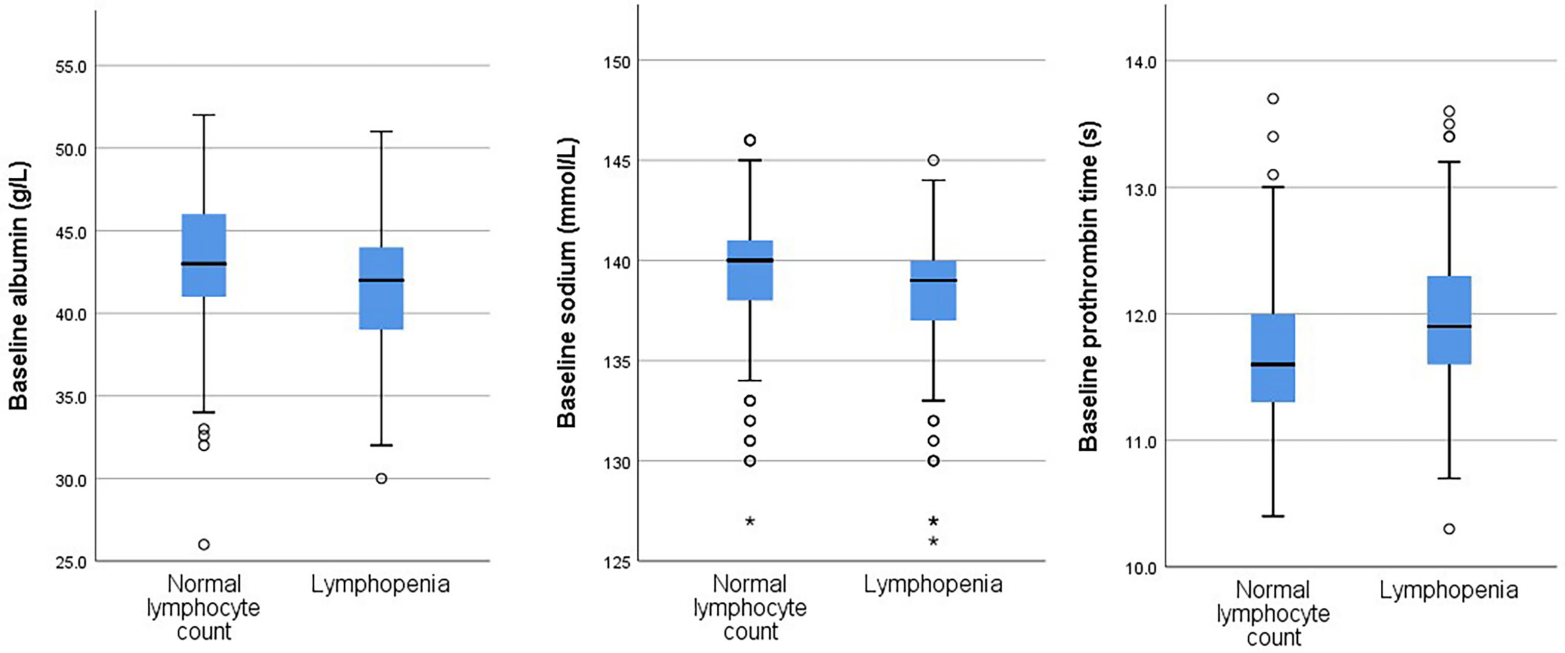
Distributions of values of baseline albumin levels, sodium levels and prothrombin times in the groups with and without lymphopenia. An extreme outlier is indicated by an asterisk.

We compared patients with normal lymphocyte count to those with lymphopenia (**Table 1**). Of note, patients with lymphopenia were older than those with normal lymphocyte count. Hence, subsequent comparisons were corrected for age. Patients with lymphopenia had lower TSH, fT4 and fT3 levels than those with normal lymphocyte count. There tended to be more men having lymphopenia in this cohort, although the difference did not reach statistical significance (age-adjusted p=0.076). Patients with lymphopenia were more likely symptomatic on presentation and had lower SARS-CoV-2 Ct value (i.e. higher viral load) upon admission. They had worse profiles of acute phase reactants (higher CRP and lower albumin levels), worse coagulation profile and lower serum sodium levels. They were more likely to require supplementary oxygen on admission. **Figure 1** shows the distribution of albumin levels, prothrombin time and sodium levels in the group with normal lymphocyte count and that with lymphopenia.

Abnormal TFTs, falling largely into three categories, were observed in 83 patients (15.3% of the cohort), in line with the findings described in our previous publication (12). (i) Seven patients likely had pre-existing thyroid dysfunction: one patient had overt thyrotoxicosis (TSH <0.01 mIU/L, fT4 51 pmol/L, fT3 15 pmol/L) with positive anti-TPO and anti-Tg, his anti-TSHR titer was elevated at 3.6 IU/L, likely representing co-existing Graves’ disease diagnosed upon admission for acute COVID-19; six patients had subclinical hypothyroidism – three of them positive for anti-TPO. (ii) Forty-five patients had abnormal fT3 levels: 41 patients had low fT3 compatible with NTIS [38 had isolated low fT3, 2 had concomitant low TSH, and one had concomitant mildly raised fT4 (24 pmol/L)]; 4 patients had elevated fT3 where assay interference could not be totally excluded [2 had isolated high fT3 (6.6 – 6.7 pmol/L); one had mildly elevated fT4 (25 pmol/L) and fT3 (6.6 pmol/L); one patient had mildly elevated TSH (5.6 mIU/L), normal fT4 (17 pmol/L) and mildly elevated fT3 (7.0 pmol/L)]. (iii) 31 patients were considered to have thyroid dysfunction compatible with different phases of thyroiditis: 25 patients had a biochemical picture compatible with subclinical thyrotoxicosis, i.e. isolated low TSH with normal fT4 and fT3; 2 patients had isolated low fT4 (11 pmol/L); 4 patients had isolated elevated fT4 (24 pmol/L).

### Variables Associated With Lymphocyte Counts

We studied the correlations of TSH, thyroid hormones and other clinical variables with the lymphocyte counts. (**Table 2**). TSH, fT4 and fT3 levels positively correlated with the lymphocyte counts. Clinical parameters having positive correlations with the lymphocyte counts included Ct value, albumin, platelet, sodium and eGFR, while those having inverse correlations with the lymphocyte counts included age, CRP, prothrombin time (PT), urea, aspartate aminotransferase (AST), lactate dehydrogenase (LDH) and creatine kinase (CK).

**Table 2:** Pearson correlation of clinical parameters with lymphocyte counts.

	Crude r	P value
TSH (mIU/L)^a^	0.231	**<0.001**
fT4 (pmol/L)	0.146	**0.001**
fT3 (pmol/L)	0.338	**<0.001**
Age (years)	-0.310	**<0.001**
**Viral load**		
Cycle threshold value at baseline^a^	0.378	**<0.001**
**Acute phase reactants**		
C-reactive protein (mg/dL)^a^	-0.308	**<0.001**
Albumin (g/L)	0.231	**<0.001**
**Coagulation profile**		
Platelet (x 10^9^/L)^a^	0.406	**<0.001**
Prothrombin time (s)	-0.199	**<0.001**
**Biochemical parameters**		
Sodium (mmol/L)	0.298	**<0.001**
Potassium (mmol/L)	0.047	0.277
Urea (umol/L)^a^	-0.180	**<0.001**
eGFR^a^	0.318	**<0.001**
AST (U/L)^a^	-0.153	**<0.001**
ALT (U/L)^a^	0.048	0.266
LDH (U/L)^a^	-0.110	**0.011**
Creatine kinase (U/L)^a^	-0.136	**0.001**

Data are presented as median (interquartile range) and number (percentage) as appropriate.

Values in bold represent statistical significance.

TSH, thyroid stimulating hormone; fT4, free thyroxine; fT3, free triiodothyronine; eGFR, estimated glomerular filtration rate; ALT, alanine aminotransferase; AST, aspartate aminotransferase; LDH, lactate dehydrogenase.

a logarithmically transformed before analysis.

Regarding categorical variables, lymphocyte counts were lower among patients with hypertension (p=0.006), malignancy (p=0.014), and elevated Troponin T (p=0.009). Lymphocyte counts did not differ according to the presence of diabetes (p=0.234), obesity (p=0.234), ischemic heart disease/heart failure (p=0.158) or lung disease (p=0.119). There was a trend towards lower lymphocyte counts among men, although the difference was not statistically significant [1.16 x 10^9^/L (IQR: 0.85 – 1.61) in men vs 1.25 x 10^9^/L (IQR: 0.94 – 1.71) in women, p=0.086]. However, patients requiring supplementary oxygen on admission, or those symptomatic on presentation had lower lymphocyte counts (both p<0.001). Lymphocyte counts did not differ when classified according to symptom burden (<3 vs ≥3 symptom counts) (p=0.128).

### TSH and fT3 Levels Were Independently Associated With Lymphocyte Counts

In the multivariable stepwise linear regression analysis (**Table 3**), among the components of TFTs, TSH (standardized beta 0.160, p<0.001) and fT3 (standardized beta 0.094, p=0.023), but not fT4, remained independently and positively correlated with lymphocyte counts. Other independent variables associated with lower lymphocyte counts included: older age, lower Ct value, higher CRP, worse coagulation profile (lower platelet and higher PT), lower sodium levels and more severe clinical presentations (symptomatic presentation and oxygen requirement on admission). On the other hand, LDH levels positively correlated with lymphocyte counts. Further inclusion of sex did not modify the independent correlation of TSH and fT3 with lymphocyte counts. Sex was not an independent determinant of lymphocyte counts.

**Table 3:** Independent determinants of lymphocyte counts on multivariable stepwise linear regression analysis.

	Standardized Beta	P value
Thyroid stimulating hormone (mIU/L)^a^	0.160	<0.001
Free triiodothyronine (pmol/L)	0.094	0.023
Age (years)	-0.132	0.001
Cycle threshold value^a^	0.208	<0.001
C-reactive protein (mg/dL)^a^	-0.165	<0.001
Platelet (x 10^9^/L)^a^	0.226	<0.001
Prothrombin time (s)	-0.090	0.010
Sodium (mmol/L)	0.085	0.026
Lactate dehydrogenase (U/L)^a^	0.134	0.002
Oxygen requirement on admission	-0.103	0.005
Symptomatic presentation	-0.093	0.011

Model included thyroid stimulating hormone, free thyroxine, free triiodothyronine, age, cycle threshold value, C-reactive protein, albumin, platelet, prothrombin time, sodium, urea, estimated glomerular filtration rate, aspartate aminotransferase, lactate dehydrogenase, creatine kinase, elevated troponin T, hypertension, malignancy, supplementary oxygen on admission, symptomatic presentation.

a logarithmically transformed before analysis.

### Sensitivity Analyses

We repeated the analyses after exclusion of patients with possibly pre-existing thyroid disorders – one patient with overt thyrotoxicosis and 6 patients with subclinical hypothyroidism. Similar results were obtained: both TSH (standardized beta 0.138, p<0.001) and fT3 (standardized beta 0.126, p=0.006) remained independently associated with lymphocyte counts in the multivariable stepwise linear regression analysis.

We evaluated the correlation between TSH and lymphocyte counts among the subgroup of 41 patients with NTIS, characterized by low fT3. TSH still showed a significant positive correlation with lymphocyte counts (*r* = 0.344, p=0.032).

### Recovery of TFTs and Lymphocyte Counts

A subgroup of patients received reassessment of lymphocyte counts and TFTs in 1 – 2 weeks’ time: 40 patients had reassessment after a median of 9 days (IQR: 4 – 15). Paired comparisons (**Table 4**) showed similar trends of improvement for TSH (p=0.031), fT3 (p<0.001) and lymphocyte counts (p<0.001), while fT4 showed no significant changes (p=0.186). Of 18 patients (45.0%) with lymphopenia on admission for COVID-19, 8 remained lymphopenic upon reassessment. Hence, 10 of 18 patients (55.6%) recovered. On the other hand, of 10 patients (25.0%) had abnormal TFTs on admission for COVID-19, 2 remained abnormal upon reassessment. Hence, 8 of 10 patients (80.0%) with abnormal TFTs had recovered.

**Table 4:** Thyroid function tests and lymphocyte counts of patients who had reassessment around 1–2 weeks after acute COVID-19 (n=40).

	Baseline	Reassessment	P value
Lymphocyte count (x 10^9^/L)^a^	1.14 (0.80 – 1.41)	1.57 (1.30 – 2.10)	**<0.001**
Thyroid-stimulating hormone (mIU/L)^a^	1.20 (0.61 – 1.60)	1.43 (0.83 – 1.78)	**0.031**
Free thyroxine (pmol/L)	17.0 (14.0 – 20.0)	17.5 (16.3 – 19.5)	0.186
Free triiodothyronine (pmol/L)	4.1 (3.7 – 4.8)	4.9 (4.5 – 5.2)	**<0.001**

Data presented as median (interquartile range).

Values in bold represent statistical significance.

a logarithmically transformed before analyses.

### Prognostic Implications of NTIS and Lymphopenia in COVID-19

Among all 541 patients, 42 (7.8%) had severe COVID-19 outcomes. When classified according to presence of lymphopenia and NTIS, there was a significant increasing trend of likelihood of severe COVID-19 outcomes with increasing number of abnormalities (p<0.001): 16 out of 332 patients (4.8%) with normal lymphocyte count and no NTIS; 17 out of 184 patients (9.2%) with either lymphopenia or NTIS; and 9 out of 25 patients (36.0%) with both lymphopenia and NTIS.

We further investigated whether NTIS or lymphopenia carried independent prognostic implications in COVID-19. The comparison between patients who did and did not develop severe COVID-19 outcomes is summarized in **Table 5**. Patients who developed severe COVID-19 outcomes were older, more likely to be men and more likely to have pre-existing cardiometabolic comorbidities. Apart from NTIS and lymphopenia, differences were observed in a range of biomarkers: a more adverse profile was noted in patients who developed severe COVID-19 outcomes. To examine whether NTIS or lymphopenia were independently associated with severe COVID-19 outcomes, we employed multivariable stepwise logistic regression analysis. In the final model of the multivariable logistic regression (**Table 6**), NTIS (adjusted OR 3.64, p=0.005) joined other known risk factors of severe COVID-19 outcomes (male, comorbidities, higher viral loads and higher inflammatory index) to be the independent variables associated with severe COVID-19 outcomes, while lymphopenia was no longer an independent predictor.

**Table 5:** Comparison between patients who did and did not develop severe COVID-19 outcomes.

	Patients without Severe COVID-19 Outcomes	Patients with Severe COVID-19 Outcomes	P value
Number	499 (92.2%)	42 (7.8%)	---
NTIS	28 (5.6%)	11 (26.2%)	<0.001
Lymphopenia	171 (34.3%)	24 (57.1%)	0.003
Age >50 years	233 (46.7%)	32 (76.2%)	<0.001
Male	217 (43.5%)	28 (66.7%)	0.004
**Comorbidities**			
Hypertension	98 (19.6%)	16 (38.1%)	0.005
Diabetes	73 (14.6%)	14 (33.3%)	0.002
Obesity	21 (4.2%)	5 (11.9%)	0.025
IHD/CHF	17 (3.4%)	5 (11.9%)	0.007
Stroke/TIA	9 (1.8%)	4 (9.5%)	0.013
Cancer	15 (3.0%)	2 (4.8%)	0.634
Symptomatic presentation	343 (68.7%)	37 (88.1%)	0.008
Ct value <25	247 (49.5%)	32 (76.2%)	<0.001
Elevated CRP	204 (40.9%)	34 (81.0%)	<0.001
Hypoalbuminaemia	65 (13.0%)	15 (35.7%)	<0.001
Thrombocytopenia	99 (19.8%)	12 (28.6%)	0.178
Elevated PT	2 (0.4%)	0 (0%)	0.999
Hyponatraemia	53 (10.6%)	13 (31.0%)	<0.001
Hypokalaemia	173 (34.7%)	12 (28.6%)	0.424
Elevated urea	15 (3.0%)	6 (14.3%)	<0.001
eGFR <60 mL/min	18 (3.6%)	5 (11.9%)	0.010
Elevated ALT	72 (14.4%)	9 (21.4%)	0.222
Elevated AST	117 (23.4%)	20 (47.6%)	0.001
Elevated LDH	165 (33.1%)	27 (64.3%)	<0.001
Elevated CK	52 (10.4%)	10 (23.8%)	0.009
Elevated troponin T	41 (8.2%)	9 (21.4%)	0.005

NTIS, non-thyroidal illness syndrome; IHD, ischaemic heart disease; CHF, congestive heart failure; TIA, transient ischaemic attack; Ct, cycle threshold; CRP, C-reactive protein; PT, prothrombin time; eGFR, estimated glomerular filtration rate; ALT, alanine aminotransferase; AST, aspartate aminotransferase; LDH, lactate dehydrogenase; CK, creatine kinase.

**Table 6:** Variables associated with severe COVID-19 outcomes in the final model of the multivariable stepwise logistic regression analysis.

Variables	Adjusted OR (95% CI)	P-value
NTIS	3.64 (1.49 – 8.91)	**0.005**
Male (vs female)	2.20 (1.05 – 4.61)	**0.037**
IHD/CHF	3.23 (0.91 – 11.5)	0.070
Stroke/TIA	5.49 (1.32 – 22.9)	**0.019**
Ct value <25	3.34 (1.47 – 7.58)	**0.004**
Elevated CRP	3.70 (1.51 – 9.10)	**0.004**
Hypoalbuminaemia	2.12 (0.92 – 4.89)	0.078
Elevated creatine kinase	2.28 (0.95 – 5.45)	0.065

NTIS, non-thyroidal illness syndrome; IHD, ischaemic heart disease; CHF, congestive heart failure; TIA, transient ischaemic attack; Ct, cycle threshold; CRP, C-reactive protein.

The model included NTIS, lymphopenia, age >50 years, male, hypertension, diabetes, obesity, IHD/CHF, stroke/TIA, symptomatic presentation, Ct value <25, hypoalbuminaemia, hyponatraemia, elevated urea, estimated glomerular filtration rate <60 mL/min, elevated aspartate aminotransferase, elevated lactate dehydrogenase, elevated creatine kinase and elevated troponin T.

Values in bold represent statistical significance.

## Discussion

Our study showed that in COVID-19, both TSH and fT3 positively correlated with lymphocyte counts independent of demographics, comorbidities, viral load, inflammatory markers and organ dysfunction. Most of the abnormal TFTs and lymphopenia recovered soon after acute COVID-19. These findings would suggest possible interactions between the hypothalamic-pituitary-thyroid axis and the immune system in COVID-19. COVID-19 patients who had both NTIS (characterized by low fT3) and lymphopenia were more likely to have severe COVID-19 outcomes compared to those who only had either one of NTIS or lymphopenia. Furthermore, NTIS, but not lymphopenia, was an independent predictor of severe outcomes in COVID-19, suggesting thyroid function to be one of the better markers of COVID-19 severity.

### Independent Associations of TSH and fT3 With Lymphocyte Counts

In this cohort of patients with predominantly non-severe COVID-19, we reported a 36.0% prevalence of lymphopenia, consistent with the rates of lymphopenia among non-severe COVID-19 patients described in the literature (varying from 1% to 80%) (2). We observed an independent positive correlation of TSH and fT3 levels with lymphocyte counts in this study. While both thyroid function and lymphocyte counts may simply be markers of illness, the fact that a significant association between thyroid function and lymphocyte counts remained after adjusting for a range of COVID-19-related parameters may suggest a potential interaction between hypothalamic-pituitary-thyroid axis and the immune system. *In vitro* incubation of T-lymphoma mouse cell line with thyroid hormones for 24 to 72 hours showed increased proliferation, mediated by protein kinase C and involved activation of inducible nitric oxide synthase (8). Hypothyroidism in humans and experimentally-induced hypothyroidism in rats have been shown to be associated with diminished thymic activity, effects that were reversed with thyroid hormone replacement (26). Reversal of propylthiouracil-induced hypothyroidism in mice with T3 replacement led to recovery of lymphocyte proliferative ability (27). Nonetheless, these results should be applied to our study with caution as TFTs in experimentally-induced hypothyroidism were characterized by low fT4 and fT3 but high TSH, in contrast to the low fT3 and low TSH concerning our study. In addition to thyroid hormones, TSH has also been shown to interact with the immune system. TSH receptors are found on the surface of B and T lymphocytes. In murine model, there was improvement in the proliferative capacity and natural killer cell activity of spleen lymphocytes by TSH (9). A study of athyreotic patients due to total thyroidectomy for differentiated thyroid cancer showed that administration of recombinant human TSH led to a significant rise in the percentage of natural killer T cells and B lymphocytes in their peripheral blood. This showed a potential direct impact of TSH on immune cells, independent of thyroid hormone action (28). Indeed, in our subgroup analysis, TSH still positively correlated with lymphocyte counts among patients with low fT3, further highlighting the independent association between TSH and lymphocyte counts.

Of note, fT4 levels did not show an independent correlation with lymphocyte count in the multivariable linear regression model. It may be because T3 is the active form of thyroid hormone converted from T4. On the other hand, it may reflect that in NTIS, fT3 and TSH drop earlier than fT4. Interestingly, a recent meta-analysis pooling 58 studies of correlations between thyroid function and clinical parameters indicates that fT4 seems to correlate with clinical parameters better than TSH and fT3 (29). There are also suggestions in the meta-analysis that correlations of TSH and fT3 with clinical parameters may be confounded by reverse causation. In our current association study, elements of reverse causality in the correlation of TSH and fT3 with lymphocyte counts could not be entirely excluded. This issue can be better answered by interventional studies on the benefits of thyroid hormone replacement in the context of lymphopenia in COVID-19 (30). Furthermore, considering the strength of the effects of TSH and fT3 on lymphocyte counts in the multivariable model, it is likely that thyroid function is only one of the many contributors to lymphopenia in COVID-19, rather than playing a dominant role, given the evidence of expression of the entry receptor of SARS-CoV-2, ACE2, in various human tissues (14).

### Other Independent Determinants of Lymphocyte Counts in COVID-19

Our study revealed multiple independent determinants of lymphocyte counts in COVID-19 (2). Lymphocyte counts decline with age, consistent with other studies (31), which may be related to thymic involution leading to changes in the overall immune competence (32). Secondly, SARS-CoV-2 PCR Ct values positively correlated with lymphocyte counts, meaning that a higher SARS-CoV-2 viral load is associated with lymphopenia. This suggested a viral-specific mechanism of lymphopenia. Indeed, expression of ACE2, the entry receptor for SARS-CoV-2, has been found in lymphocytes. Hence, there may be a direct cytotoxic effect from SARS-CoV-2 (1, 33). Thirdly, higher levels of inflammatory markers such as CRP were associated with lymphopenia. Interleukin 6 (IL-6) is known to induce gene expression and release of CRP from the liver and from immune cells (34). IL-6 is highly expressed during viral infection, and can cause apoptosis of lymphocytes (35). Fourthly, the positive correlation between platelet and lymphocyte counts suggested a possible element of infection of the bone marrow resulting in abnormal hematopoiesis (36). Fifthly, the association of lower platelet counts, increasing PT and oxygen requirement with lower lymphocyte counts could be explained by the COVID-19-related cytokine storm leading to disseminated intravascular coagulopathy and acute respiratory distress syndrome (37). These proinflammatory cytokines can suppress the lymphocyte proliferation. Lastly, the association between hyponatremia and lymphopenia could be explained by the increase in proinflammatory cytokines in COVID-19. IL-6 may provide a common link between hyponatremia and lymphopenia, as IL-6 can lead to lymphopenia and has been shown to be inversely correlated with sodium levels in COVID-19 in an Italian study (38). In that study, hyponatremia improved after administration of tocilizumab, an IL-6 receptor antagonist (38). IL-6 may play a pathogenic role in causing electrolyte disturbance by inducing non-osmotic release of vasopressin (39).

### Thyroid Function and Lymphocyte Counts With Recovery From COVID-19

The trajectories of thyroid function recovery suggest that COVID-19 is the cause of the thyroid abnormalities. A recent study which evaluated a Dutch cohort of COVID-19 patients reported the comparison of TSH, thyroid hormones and inflammatory markers between 17 patients with severe lymphopenia and 18 patients without lymphopenia (15). In line with their findings, we found that patients with lymphopenia had lower TSH, fT4 and fT3, and higher CRP levels. Furthermore, we revealed the independent association of TSH and fT3 with lymphocyte counts even after adjusting for the levels of acute phase reactants. Interestingly, in that Dutch cohort, among the 15 COVID-19 patients who underwent reassessment blood tests 1 week later, 12 of them showed recovery of lymphocyte counts approaching normal ranges, whereas thyroid hormones did not significantly change, especially T3 levels remaining relatively low. The authors thus concluded that the results argued either for different kinetics of recovery of lymphopenia and thyroid function, or against a direct causal relationship between lymphopenia and thyroid function abnormalities. Among our 40 patients who underwent reassessment of TFTs and lymphocyte counts, we observed a parallel recovery in TSH and fT3 with lymphocyte counts. The differences between our results and those from the Dutch cohort could be explained by the milder spectrum of COVID-19 in our cohort, and the differences in inclusion criteria in the Dutch cohort. Our results could support a different kinetics in the recovery of thyroid function and lymphopenia, such that in milder cases, thyroid function and lymphopenia may recover in parallel. Nonetheless, our results may still be consistent with a possible direct interaction between the hypothalamic-pituitary-thyroid axis and the immune system. Given that this is an association of our study, whether thyroid hormone replacement is beneficial in the context of lymphopenia remains to be elucidated in ongoing studies in COVID-19 (30).

### Prognostic Implication of Thyroid Function

Our study revealed that patients with NTIS, but not lymphopenia, would have worse COVID-19 outcomes. Among COVID-19 patients with NTIS, low TSH also held prognostic significance. SARS-CoV-2 infection and its associated inflammation can lead to both lymphopenia and NTIS, so thyroid function and lymphopenia may merely reflect COVID-19 severity, where our results might suggest that thyroid function is among the better markers of COVID-19 severity, instead of lymphopenia. On the other hand, pre-clinical studies have demonstrated the influence of thyroid hormones and TSH on lymphocyte counts. As lymphopenia is believed to be a defective immune response to the virus (40), such influence on lymphocyte count may contribute to the prognostic significance of NTIS and low TSH in the context of NTIS.

Male sex has been described in different populations to be associated with worse COVID-19 outcomes including mortality (41). Hence, it might be expected to observe a male predominance among the group with lymphopenia, a marker of COVID-19 severity. In line with this, in our study, we observed a trend towards more men having lymphopenia, and lymphocyte counts tended to be lower in men than in women. Moreover, male sex was among the five prognostic factors for severe COVID-19. Nonetheless, as only total lymphocyte counts were measured in our study, further details about lymphocyte subsets were not available. Some studies have demonstrated differences in the patterns of lymphocyte subsets which may explain the worse prognosis of COVID-19 among men (42). This may explain the lack of significant sex bias observed in the current study.

Our study shed light onto the interaction between TSH/thyroid hormones and the immune system. It also offered a potential explanation for the prognostic role of NTIS in COVID-19. Our results were generated from a relatively large cohort of complete thyroid function assessment, thus allowing adjustments for multiple potential confounders. Nevertheless, our study should be interpreted bearing the following limitations. Firstly, this is an observational study of associations between TFT and lymphocyte count, which do not prove causality. Secondly, TFTs were only reassessed one week after the initial TFTs on admission. Further studies with more frequent TFT monitoring during the course of illness can delineate the kinetics of TFT and lymphocyte count recovery with a higher resolution. Thirdly, SARS-CoV-2 viral loads were represented by Ct values. Despite a good correlation (23, 24), direct quantitative measurements of viral loads would have been preferable if available. Fourthly, obesity was defined by the ICD-9-CM diagnostic code in our study as a categorical variable, instead of body mass index as a continuous variable, and was likely to be underreported. Fifthly, high-resolution computed tomography was done at the physicians’ discretion. Thus, the detection of imaging features of pneumonia in our cohort might be less sensitive. Last but not least, only total lymphocyte counts were measured in this study. Further details about lymphocyte subsets were not available, which may provide more insights into the interrelationship between the hypothalamic-pituitary-thyroid axis and the immune system.

## Conclusion

TSH and fT3 levels showed independent positive correlations with lymphocyte counts among COVID-19 patients. There was a parallel recovery in TFTs and lymphocyte count around 1 week after acute illness. These results suggested potential interactions between the hypothalamic-pituitary-thyroid axis and the immune system. Furthermore, patients with both lymphopenia and NTIS had the worst clinical course of acute COVID-19, supporting the potential prognostic role of thyroid hormones in COVID-19.

## Data Availability Statement

The raw data supporting the conclusions of this article will be made available by the authors, without undue reservation.

## Ethics Statement

The studies involving human participants were reviewed and approved by the institutional review board of the University of Hong Kong/Hospital Authority Hong Kong West Cluster. The patients/participants provided their written informed consent to participate in this study.

## Author Contributions

DL wrote the manuscript. DL, CHL, WC, AL, AT, PP, TH, CC, and CYL researched the data. DL and CF performed statistical analyses. CHL, WC, AL, KKT, CWL, KCT, YW, IH, and KL critically reviewed and edited the manuscript. KL initiated and supervised the study, is the guarantor of this work, has full access to all the data in the study and takes responsibility for the integrity of the data and the accuracy of the data analysis. All authors contributed to the article and approved the submitted version.

## Conflict of Interest

The authors declare that the research was conducted in the absence of any commercial or financial relationships that could be construed as a potential conflict of interest.

## Publisher’s Note

All claims expressed in this article are solely those of the authors and do not necessarily represent those of their affiliated organizations, or those of the publisher, the editors and the reviewers. Any product that may be evaluated in this article, or claim that may be made by its manufacturer, is not guaranteed or endorsed by the publisher.
